# Stochastic synchronization of neurons: the topologicalimpacts

**DOI:** 10.6026/97320630014504

**Published:** 2018-12-09

**Authors:** Saurabh Kumar Sharma, Md. Zubbair Malik, R.K Brojen Singh

**Affiliations:** 1 School of Computational and Integrative Sciences, Jawaharlal Nehru University, New Delhi-110067, India

**Keywords:** neurons organization, Hindmarsh-Rose, Synchronization, amplified signal

## Abstract

Cross-talk among coupled stochastic Hindmarsh-Rose (HR) neurons is significantly affected by the topology of the neurons organization. If the coupled stochastic HR neurons are arranged in the form of ring topology with odd number of neurons, the neurons are in anti-phase synchronization with homogeneous distribution of phase ordering of the oscillators. On the other hand, if the coupled HR oscillators are arranged in the ring topology with even number of oscillators, the oscillators are formed into two groups which are anti-phase synchronized, but all the oscillators in each group are in in-phase synchronization.Synchronization of the HR oscillators due to coupling in all topological arrangements is affected by the noise.However, noise can induce optimal coherence of the cross-talked oscillators at a particular value at which signal processing is the most favorable with amplified signal, the phenomenon known as stochastic resonance.

## Background

Communication among coupled natural systems could be the origin of emergence of important local and global properties, starting from normal state to chaos, crises, chimera and many other peculiar states. One way to deal with such complicated communication among the systems is to study synchronization among these modeled systems using various coupling mechanisms and explore all possible exhibited properties 1-5. Further, since noise is an inherent parameter in natural systems, it plays an important role in terms of hindrance in signal processing to protect themselves from unwanted external signals (e.g. disease signal, cancer wave, irradiation etc.) and constructive way (enhance signal detection, amplification of signal etc) known as stochastic resonance 6,7. On the other hand, topology of the coupled rhythmic systems also affects the properties of the synchronization 8 which lacks intensive study in this direction. Since mechanisms in living systems are noise driven processes, noise helps in various ways 9-13), starting from molecular to phenotypic level: to survive, stay fit, and for protection from the competing environment. For example, pathogens use noise to create phenotype diversities to enable to survive in the host 14; higher level organisms use it for adaptation 15,16; cells use it to make important decisions and their fates 9 and various cellular phenotypes 17.

The processes in neuron dynamics comprise of random interaction of ions *(Na^+^,K^+^,Ca^2+^)* 18,19, random closing and opening of ion channels 20, and synaptic inputs from the surrounding neurons 21 and can be modeled using stochastic framework 22. These stochastic processes trigger random firing of membrane potential and other related variables 23. Since chaotic nature is one of the important features in brain states, Hindmarsh–Rose (HR) model 19, which is a modified version of Fitzhugh model 24, can be taken as a significant model because the can generate bursting spiking patterns and chaotic behavior that is closely mimic to various physiological states of brain. Even though stochastic formalism of this model is not straightforward, one can model HR model using chemical Langevin equation (CLE) formalism 25. There have been modifications in CLE formalism based on maintaining positivity of the noise part 26 and complex CLE formalism 27, but these modifications either missing significant contributions from the system variables or limited to some specific models. One way to rescue from this unphysical meaning of negative molecule numbers is to rescale the variables in the model equations 28 and can construct a meaningful stochastic theory of HR model.

The effect on synchronization by the topology of the coupled HR oscillators is the variation in synchronization threshold of the coupled oscillators 8. Cross-talk among the neurons can be well studied using the concept of synchronization 2 and is a well-studied phenomenon in deterministic HR model using various coupling mechanisms like electrical 29,30 and chemical coupling 31, non-local coupling 8,8, and memristor synaptic coupling 32. However, the impact of topology of the coupling neurons and its interplay with noise on the neuron communication are not fully studied.The emergence of interesting phenomena of communication through coherence (CTC) is a phenomenon of neuronal communication through interaction of large number of neurons in a network in brain from the complicated neuronal network might be triggered by the topology of the coupled neurons. We focus on the issue of how topology of the interacting neurons affects the properties of communication among these coupled neurons.

## Methodology

Please see supplementary material for Methodology.

## Results and Discussion

Topology of arrangement of the coupled identical HR oscillators affects in various ways. The coupled HR oscillators are arranged in a ring with environmental coupling mechanism assigned among them and coupling is done via slow current due to, *Ca^2+^* (z)variable and look for possible synchronization among the remaining variables x and y. When the coupled oscillators exhibit in-phase synchronization, the threshold synchronization value (the minimum value of the coupling constant ε at which the coupled oscillators just exhibit synchronization) increases as the number of coupled oscillators is increased similar to the reported work in 8. Noise in the coupled HR systems, however, affects significantly in the rate of synchronization by allowing to increase the threshold synchronization value, which means coupled stochastic HR oscillators need higher value of the threshold synchronization value to exhibit synchronization.

**Figure 1:**
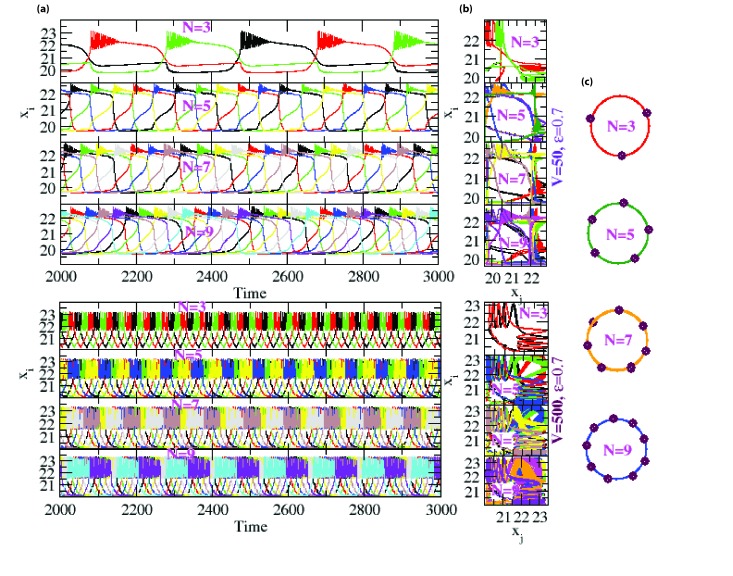
Anti-phase synchronization among odd number of coupled HR oscillators in a ring topology: (a) Dynamics of anti-synchronized xi, i = 3, 5, 7, 9 of coupled HR oscillators for V = 50, epsilon = 0.7 and V = 500, epsilon = 0.7 respectively (left column); (b) Recurrence plots of the coupled HR oscillators showing anti-phase synchronization (second column); (c) Arrangement of coupled HR oscillators with phase distribution (right column).

**Figure 2:**
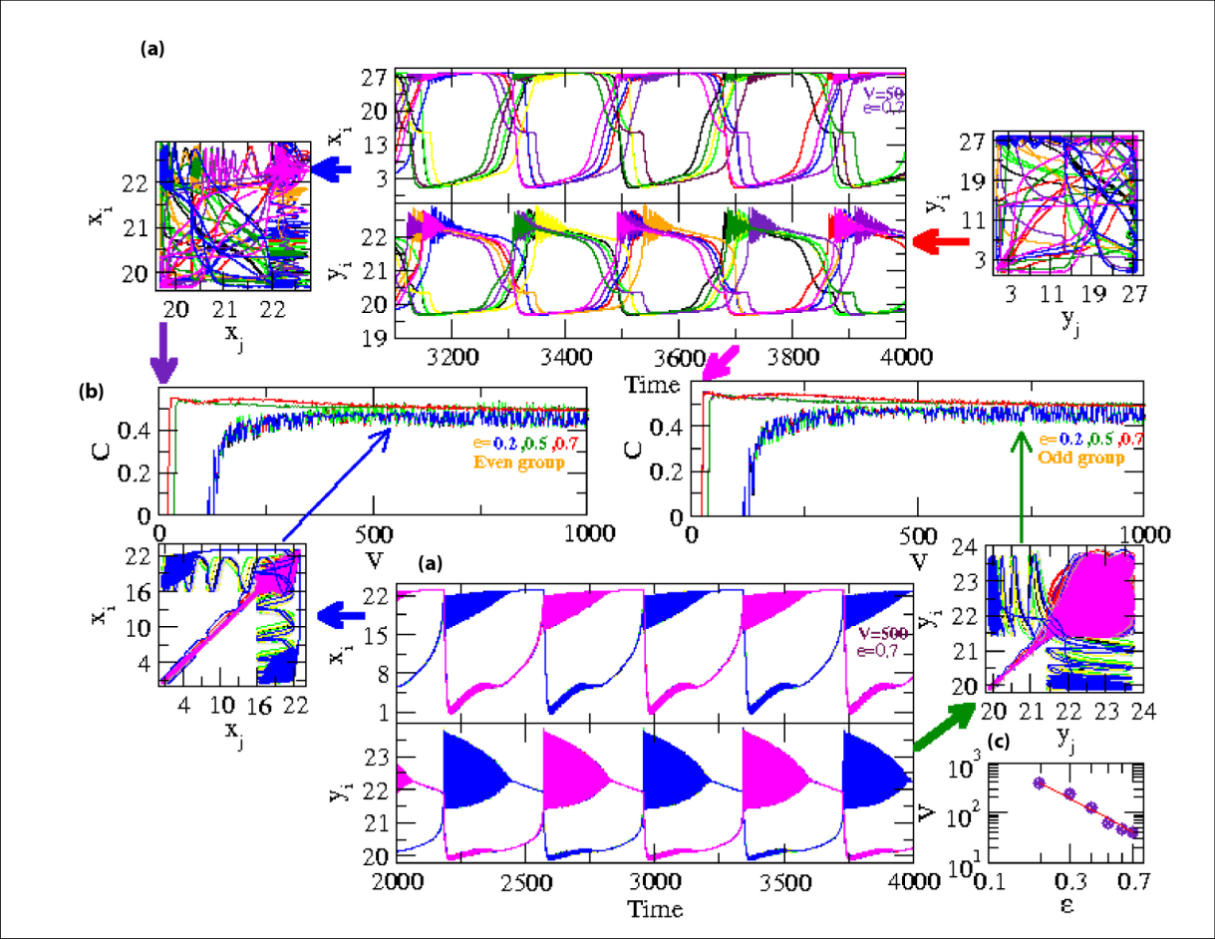
Synchronization of coupled HR oscillators arranged in the form of ring with even number of oscillators: (a) Dynamics of xi and yi at V = 500 with epsilon values 0.2 and 0.7 respectively (upper and lower panels) with corresponding recurrence plots; (b) Correlation function as a function of V calculated at epsilon = 0.2, 0.5, 07 respectively (middle row); (c) Plots of V as a function of Î (lower right corner panel).

The scenario of synchronization is in different way when the coupled HR oscillators are in anti-phase synchronization and topology of the oscillators play an important role in achieving synchronization. When the number of coupled oscillators is odd, the anti-phase synchronization takes place in such a way that the HR oscillators are arranged by distribution of the oscillators at equal phases (Figure 1). If the coupled number of oscillators is three (N = 3), then at V = 50 the oscillators will achieve anti-phase synchronization at ε = 0.7 with phases 2π/3, so on, such that for N=N_0_ (odd) and for the same parameter values, the coupled HR oscillators will be in anti-phase synchronization with each oscillator at the phase θN_0_=2π⁄N_0_ (Figure 1). We then increased the value of *V* i.e. by decreasing noise strength (noise η α 1/√*V*) by taking V = 500 with same ε, the coupled HR oscillators follows the same trend of anti-phase synchronization with θ_N_0__=2π⁄*N_0_* phase distribution of each synchronized oscillators. In this case strength of synchronization of the coupled HR oscillators is more than the lower value of *V* i.e. stronger noise system. The anti-synchronization phenomenon is shown by recurrence plot (see the materials and methods) with points along second diagonal (Figure 1 second column) and correlation plot with V (Figure 2 middle row). Now, if the number of oscillators is even, the way how the coupled HR oscillators exhibit anti-phase synchronization is quite different from the way how odd number of coupled HR oscillators exhibit anti-phase synchronization. In this case, half of the total oscillators N_e_/2 are in in-phase synchronization, whereas, the other half of the oscillators again shows in-phase synchronization, but these two groups exhibit anti-phase synchronization (Figure 2) with each other.

These in-phase and anti-phase synchronization phenomena are detected by recurrence plots in (x_i_, x_j_) and ((y_i,y_j) ∀i, j = 1, 2, ...,N (for in-phase synchronization distributions of phase points are along the diagonal, whereas, for anti-phase synchronization the phase points are along the opposite diagonal), and correlation C with V plots (Figure 2). The way how the N_e_ oscillators are distributed among the two groups is as follows: the alternate oscillators are in the same group, and the two groups are anti-synchronized with phase = π. 

The impact of noise in the rate of synchronization of coupled HR oscillators is quite significant. The coupled HR oscillators could not able to exhibit complete synchronization (C→1) because noise fluctuations which can be measured from V. For small value of coupling constant ε=0.2 the oscillators show strong synchronization (both in-phase and out-phase synchronization) at large values of V i.e. significantly low noise in the system. But for significantly large values of ε (ε ≥0.5), a peculiar scenario can be seen in the synchronization behavior of the oscillators. The oscillators attains strong synchronization at a particular value of V for a fixed ε, and then decreases as V increases. This means that C→_max_ (C_max_ is maximum correlation value for a particular ε and noise strength V_0_, but C < C_max_ for both V>V_0_ and V<V_0_, which could be the case of stochastic resonance 6,7, where signal processing of the coupled HR oscillators show maximum at V→V_0_ (Figure 2 second row panels). Similar scenario can be seen both in even and odd groups of coupled HR oscillators. Further, the threshold coupling parameter value varies as a function of noise in the system V and follows power law behavior V ~ ε ^−α,^ where, α is power law exponent with value α=2.3.

The synchronization among a coupled stochastic HR oscillator in a certain topology via environmental coupling mechanism is significantly affected by the topology of the network driven by both noise in the system and coupling parameter. When the topology of the oscillators in the ring is odd, each individual in the topology might not able to arrange in grouping the oscillators, and each individual become anti-phase synchrony to every other oscillator. However, if the number of oscillators is even, they might able to group into two which exhibit anti-phase synchronization. Further, noise could able to show maximum synchronization at a particular *V* value which could be the case of stochastic resonance.

## Conclusion

The signal processing among the neurons is affected by various factors and parameters, more importantly by topology of the oscillators under environmental coupling mechanism. The arrangement of the coupled HR oscillators may trigger different way of signal processing during the cross-talk among them. When the number of coupled oscillatore is odd, grouping into two groups is not possible and hence the situation could be most favorable case for anti-phase synchronization. The oscillators in each group are in in-phase synchronization, whereas, the two groups show anti-phase synchronization. This type of environmental coupling mechanism could be quite possible in multi-neuron cross-talk which could utilize the optimization of signal processing depending on the topology the neurons network organization to exhibit coherence patterns of cortical neurons in brain 43. Noise can be considered as an inherent fundamental parameter in natural systems which is incorporated in the system dynamics, and is very sensitive to the systems, their cross-talk and systems topology. Further, the noise in the system can be related to neuron size, which is quite variable due to both inherent internal and external fluctuations and can able to perform both in destructive and constructive ways. This noise can able to optimize the signal processing in the coupled system generally for fast information processing and signal amplification, the phenomenon known as stochastic resonance. This change in internal noise can also trigger possibilities of cross-talk mechanisms at different neuro physiological states driven by variation in neuron size (mainly axon and dendrite) 34-37. Coherence due to communication, which can be observed in brain due to interacting neurons, is highly dynamic and very sensitive to noise fluctuations 44. Moreover, the change in topology with noise in brain stimulus may trigger a drastic change in neurons communication and their functioning. Hence, rigorous theoretical and experimental studies need to be done to observe such phenomena so that one can able to understand signal processing in brain at fundamental level.

## Supplementary material

Data 1
