# Supplementary material for: Stochastic synchronization of neurons: the topologicalimpacts
Source: Bioinformation. 2018 Dec 9;14(9):504–10. doi: 10.6026/97320630014504 (PMC6563662; doi:10.6026/97320630014504)
Supplement: Data 1 [file 97320630014504S1.pdf]

BIOINFORMATION
Discovery at the interface of physical and biological sciences
OPEN ACCESS GOLD

Methodology:

Chemical Langevin equation of Hindmarsh-Rose neuron model:

Hindmarsh–Rose (HR) model **[19]** is an excitable neuron model **[33]** and can able to exhibit bursting spiking patterns as well as chaotic nature which may correspond to various neuro physiological states. The HR model system is described by,

$$\frac{d}{dt}H = R(x,y,z;t); \quad H = \begin{bmatrix} x \\ y \\ z \end{bmatrix}; \quad R(x,y,z;t) = \begin{bmatrix} y - ax^3 + bx^2 - z + I_{ex} \\ c - dx^2 - y \\ r[s(x - x_0) - z] \end{bmatrix} \quad \rightarrow (1)$$

where x, y and z are variables corresponding to membrane potential, fast current (due to either $Na^+ or\ K^+$ions) and slow current (corresponding to, Ca^{2+}), respectively. The system parameters are chosen as a= 1, b = 3, c = 1, d = 5, x_0 = −1.6, s = 4, I_{ext} = 3.25. Various parameter values of r can exhibit different patterns of the system.

The neuron dynamics can be considered as a stochastic process due to random interaction of various ions (Na^+, K^+andCa^{2+}) inside the neuron system, random diffusion of these ions through ion channels, and driving random external fluctuations. Further, various experiments show that the size of a neuron can be affected by various factors; namely,$Sar1a^R$, siRNA and siRNA + $Sar1a^R$ induced growth in dendritic and axonal lengths by 15%–45% in hippocampal neurons **[34],** organo phosphorus pesticides suppress the growth of axon and dendrite by 10%–30% in cultured sympathetic neurons of rodent **[35],** p160ROCK inhibition in cortical actin network stability causes the outgrowth of axon by 20%–40% in mammalian **[36],** and bursts of depolarizing current induced action potential and blocking of calcium current bring about rapid contraction of dendritic spine head causing variation in hippocampal neurons' size **[37].** The macroscopic variables x, y, and z can be considered as the manifestation of the mentioned complicated interaction of microscopic ions driven by random environment in the neuron. Since $x \propto \frac{Q}{r}$, with Q as total charge in the neuron, the sign in x indicates the nature of the force. Similarly, since y $\propto \frac{Q}{t}$, the sign in y shows the direction of the net charge flow in the neuron. Now, without loss of generality the variables x and y can be scaled with a scale factor α as ($x \rightarrow x + \alpha$, and $y \rightarrow y + \alpha$) to get rid of negative values involved in these variables **[28].** Following this scaling procedure, equation (1) becomes

$$\frac{d}{dt}H = R(x,y,z;t); \ H = \begin{bmatrix} x \\ y \\ z \end{bmatrix}; \ R(x,y,z;t) = \begin{bmatrix} y - ax^3 + x^2(b + 3a\alpha) - x(3a\alpha^2 + 2b\alpha) - z + a\alpha^3 + b\alpha^2 + I_{ex} \\ (c + \alpha - d\alpha^2) - dx^2 + 2d\alpha x - y \\ r[s(x - x_0) - z - s\alpha] \end{bmatrix} \quad \rightarrow (2)$$

If we keep the same values of all constants in equation (1), the scaled model (2) with α = 13, same behavior of x, y, and z can be seen in rescaled model. Now, consider the size of a neuron be V at a temperature T with well-stirred ions (Na^+, K^+, Ca^{2+}) then the resulting stochastic dynamics of state vector $H^{[s]} = [X, Y, Z]^{-1}$, where, X = xV, Y = yV, Z = zV. Each term in the three differential equations (2) corresponds to a particular state transition in the neuron given by

$$n_1X + n_2Y + n_3Z \xrightarrow{k_i} m_1X + m_2Y + m_3Z \quad \rightarrow (3)$$

where k_i is classical rate constant of ith reaction (3) with reactant and product number state vectors $n_i = [n_1, n_2, n_3]^{-1}$, $m_i = [m_1, m_2, m_3]^{-1}$, and state change vector $v_i = n_i - m_i$.

The propensity function, which can be defined as the probability that the reaction (3) can be fired anywhere in the system **[22],** can be written as $a_i = c_i \hbar_i$, where $\hbar_i$ is the possible molecular combinations and c_i is the stochastic rate constant given by $c_i = k_i V^{1-v_i}$. Hence, there are thirteen such reaction channels which are constructed from each term in the model (2) **(see Table 1) [22, 38].** Following **[25]** and defining$\Lambda_j = [H^{[s]}(t), t]$as the number of times the jth state change takes place within the time interval$[t, t + \Delta t]$, with $\Delta t > 0$; $\Delta t << 1$, chemical Langevin equation of HR model can be derived as given by,

$$H_i^{[s]}(t + \Delta t) = H_i^{[s]}(t) + \sum_{j=1}^{M} \Lambda_j \left[H^{[s]}(t), \Delta t\right] v_{jI} , \qquad where\ M = 11; \quad i = 1,2,3 \quad \rightarrow (4)$$

The equation (4) can be simplified by imposing two important approximations: (1) $\Delta t \rightarrow 0$ limit, where no significant change in state change is likely to occur, and $\Delta a_i \sim 0$. This situation can be achieved when reactant molecular population is large, and Λ_j can be approximated to Poisson random variable $\Lambda_j \rightarrow P_j(a_j, \Delta t)$. (2) $\Delta t \rightarrow$ large limit allows $a_j >> 1$, and one can approximate $P_j(a_j) \rightarrow$

©2018 BIOMEDICAL INFORMATICS

$N_j(a_j\Delta t, a_j\Delta t)$(normal random variable). These two conditions can be applied simultaneously at the large population limit [25]. Then, applying $N(m,\sigma^2) = m + \sigma N(0,1)$, where m and σ are mean and standard deviation, and putting $\frac{H_i^s(t+\Delta t)-H_i^s(t)}{\Delta t} \approx \frac{dH_i^s(t)}{dt}$, we arrive at

$$\frac{dH_i^{[s]}(t)}{dt} = \sum_{j=1}^{M} v_{ji}\, a_j\left[H^{[s]}\right] + \sum_{j=1}^{M} v_{ji}\sqrt{a_j[H^{[s]}]}\,\xi_j;\;\; \xi_j = \lim_{t\to 0} N\left(0,\frac{1}{\Delta t}\right) \qquad \rightarrow(5)$$

where ξ_j are noise parameters. Now applying the transition of states in Table 1 to equation (5), and putting x = X/V, y = Y/V, z = Z/V, we arrive at CLE for HR model,

$$\frac{d}{dt}H = R(x,y,z;t) + \frac{1}{\sqrt{V}}G(x,y,z,\xi;t); where\ H = \begin{bmatrix} x \\ y \\ z \end{bmatrix} \qquad \rightarrow(6)$$

$$R(x,y,z;t) = \begin{bmatrix} y - ax^3 + x^2(b+3a\alpha) - x(3a\alpha^2 + 2b\alpha) - z + a\alpha^3 + b\alpha^2 + I_{ex} \\ (c+\alpha-d\alpha^2) - dx^2 + 2d\alpha x - y \\ r[s(x-x_0) - z - s\alpha] \end{bmatrix}$$

$$G(x,y,z,\xi;t) = \begin{bmatrix} \sqrt{y}\,\xi_1 - \sqrt{ax^3}\xi_2 + \sqrt{x^2(b+3a\alpha)}\,\xi_3 - \sqrt{x(3a\alpha^2+2b\alpha)}\xi_4 - \sqrt{z}\,\xi_5 + \sqrt{a\alpha^3 + b\alpha^2 + I_{ex}}\xi_6 \\ \sqrt{(c+\alpha-d\alpha^2)}\xi_7 - \sqrt{dx^2}\xi_8 + \sqrt{2d\alpha x}\xi_9 - \sqrt{y}\xi_{10} \\ \sqrt{rsx}\xi_{11} - \sqrt{z}\xi_{12} - \sqrt{rsx_0 + rs\alpha}\xi_{13} \end{bmatrix}$$

The first term in equation (6) is the deterministic counterpart, and the second term is due to internal noise which scales as $1/\sqrt{V}$.

Table 1: Set of differential equations used in the model

Differential equations	Transition of states	Propensity function
$\frac{dx}{dt} = y - ax^3 + x^2(b+3a\alpha) - x(3a\alpha^2+2b\alpha) - z + a\alpha^3 + b\alpha^2 + I_{ex}$	$Y \xrightarrow{1} X$	Y
	$3X \xrightarrow{a} \phi$	$\frac{1}{3!\,V^2}aX(X-1)(X-2)$
	$2X \xrightarrow{b+3a\alpha} 3X$	$(b+3a\alpha)X(X-1)$
	$X \xrightarrow{3a\alpha^2+2b\alpha} \phi$	$X(3a\alpha^2+2b\alpha)$
	$Z + X \xrightarrow{X^{-1}} \phi$	$\frac{Z}{V}$
	$\phi \xrightarrow{a\alpha^3+b\alpha^2+I_{ex}} X$	$Y(a\alpha^3+b\alpha^2+I_{ex})$
$\frac{dy}{dt} = (c+\alpha-d\alpha^2) - dx^2 + 2d\alpha x - y$	$\phi \xrightarrow{(c+\alpha-d\alpha^2)} Y$	$Y(c+\alpha-d\alpha^2)$
	$2X + Y \xrightarrow{dY^{-1}} 2X$	$\frac{1}{2V^2}dX(X-1)$
	$X \xrightarrow{2d\alpha} X + Y$	$2d\alpha x$
	$Y \xrightarrow{1} \phi$	Y
$\frac{dz}{dt} = r[s(x-x_0) - z - s\alpha]$	$X \xrightarrow{rs} Z$	rsX
	$Z \xrightarrow{1} \phi$	Z
	$Z \xrightarrow{(srx_0+rs\alpha)Z^{-1}} \phi$	$srx_0 + rs\alpha$

Environmental coupling mechanism of Neurons:

Consider two stochastic HR neurons defined by$H^{[1]} = [x_1, y_1, z_1]^{-1}$and $H^{[2]} = [x_2, y_2, z_2]^{-1}$. When these two neurons are coupled through a common dynamic environment via membrane potential (x), the mean of the internal noise of the $x$arise from the two neurons is also being associated with the dynamics of the neurons. Then, by using this coupling scheme **[39, 40],** we have

$$\frac{d}{dt}H^{[1]} = W^{[1]} + \frac{\epsilon_1 k_1}{\sqrt{V}}\begin{bmatrix} 0 \\ 0 \\ \theta\Lambda \end{bmatrix} \qquad \rightarrow(7)$$

$$\frac{d}{dt}H^{[2]} = W^{[2]} + \frac{\epsilon_1 k_2}{\sqrt{V}}\begin{bmatrix} 0 \\ 0 \\ \theta\Lambda \end{bmatrix} \qquad \rightarrow(8)$$

$$\frac{d\theta}{dt} = -\omega\theta - \frac{\epsilon_2}{M}\sum_{i=1}^{M} k_i[x_i - \alpha]$$

$$W^{[i]} = R^{[i]} + \frac{1}{\sqrt{V}}G^{[i]};\quad H_i = \begin{bmatrix} x_i \\ y_i \\ z_i \end{bmatrix}$$

$$\Lambda = \frac{1}{M}\sum_{i=1}^{M} \Gamma_1^{[i]};\quad M = 2$$

where ω is damping factor of the decay in environmental dynamics. ϵ_1 and ϵ_2 are feedback strengths to the system and environment respectively. k_1 and k_2 are feedback adjusting parameters to the systems and environment too. Here, environment means the extracellular trafficking of ions experienced by each neuron as a consequence of surrounding neurons.

Detection of synchronization

The following two techniques are considered to detect the synchronization of two coupled systems.

Recurrence plot: If $x_1(t)$ and $x_2(t)$ are two variables corresponding to two coupled systems, then the two-recurrence plot (x_1, x_2) can able to characterize the rate of synchronization of the two coupled systems **[41].** If the points in the (x_1, x_2) plot are randomly scattered, then the two systems are uncoupled. However, if the points are concentrated along one of the diagonals of the (x_1, x_2) plot, then the systems are either in-phase synchronized, or anti-phase synchronized. The thickness of the points along the diagonal indicates how strong the synchronization can be achieved between the two coupled systems (the thinner the line the stronger is the synchronization).

Cross-correlation function:

The equal-time cross-correlation function of the two variables $x_1(\epsilon, t)$, and $x_2(\epsilon, t)$, corresponding to two coupled systems, with coupling strength ϵ, can be defined as **[42]**

$$C_{x_1,x_2}(\epsilon) = \frac{\langle x_1 x_2\rangle - \langle x_1\rangle\langle x_2\rangle}{\sigma_1\sigma_2} \quad \rightarrow(9)$$

where $\sigma_i = [\langle x_i^2\rangle - \langle x_i\rangle^2]^{1/2}$and *i*= 1, 2 are standard deviations, where $\langle \ldots \rangle$ denotes the time average. Then, this order parameter can characterize synchronization strength of the two coupled oscillators as follows:

$C_{x1,x2}$ ($\in$) = 1 (if the two systems are synchronized); = 0 (if the two systems are uncoupled); = -1 (if the two systems are anti-synchronized)

©2018 BIOMEDICAL INFORMATICS
